# Verification of sap flow characteristics and measurement errors of *Populus tomentosa* Carr. and *Salix babylonica* L. based on the liquid level equilibrium method

**DOI:** 10.3389/fpls.2022.946804

**Published:** 2022-08-30

**Authors:** Yunjie Liu, Hanhan Zhang, Changming Ma, Bingxiang Liu, Changjun Ding

**Affiliations:** ^1^College of Forestry, Hebei Agricultural University, Baoding, China; ^2^The County Forestry Development Service Center, Handan, China; ^3^State Key Laboratory of Tree Genetics and Breeding, Key Laboratory of Tree Breeding and Cultivation of State Forestry Administration, Research Institute of Forestry, Chinese Academy of Forestry, Beijing, China

**Keywords:** *Populus tomentosa* Carr., *Salix babylonica* L., sap flow, influencing factors, dissipation probe method, liquid level equilibrium method, Granier calibration

## Abstract

This study clarified the characteristics and influencing factors of sap flow in *Populus tomentosa* Carr. and *Salix babylonica* L., and verified the applicability of Granier’s original formula for measuring the sap flow of the two species, aimed to provide a basis for the accurate assessment of tree transpiration. *P. tomentosa* and *S. babylonica* were used as research objects, their sap flow was measured by the thermal dissipation probe method (TDP), together with changes in meteorological factors and soil water content. Meanwhile, the transpiration of both species was measured by the liquid level equilibrium method (LLE) to verify the applicability of Granier’s original formula. We found that: (1) the sap flow velocity of *P. tomentosa* and *S. babylonica* under typical sunny and cloudy conditions showed unimodal or bimodal changes, which were highly significantly correlated with meteorological factors (*P* < 0.01), but they were all small and poorly correlated with meteorological factors on rainy days. (2) The sap flow velocity of both species was significantly and negatively correlated (*P* < 0.05) with the daily change in stem and soil water content at 10–20 cm. (3) Compared to that calculated with the LLE method, the sap flows of the two species calculated by the TDP technique using Granier’s original formula were seriously underestimated, with error rates of -60.96% and -63.37%, respectively. The Granier’s correction formulas for *P. tomentosa* and *S. babylonica* established by the LLE method were F_*d*_ = 0.0287*K*^1.236^ (*R*^2^ = 0.941) and F_*d*_ = 0.0145*K*^0.852^ (*R*^2^ = 0.904), respectively, and the combined correction formula was F_*d*_ = 0.0235*K*^1.080^ (*R*^2^ = 0.957). It was verified that the errors of sap flow calculated by the specific correction formulas for *P. tomentosa* and *S. babylonica* were -6.18% and -5.86%, and those calculated by the combined correction formula were -12.76% and -2.32%, respectively. Therefore, the characteristics of the sap flow velocity of *P. tomentosa* and *S. babylonica* on sunny, cloudy and rainy days were different and significantly influenced by meteorological factors. The original Granier’s formula for calculating their sap flow resulted in a large error, but can be measured more accurately by constructing specific correction and combination formulas through the LLE method.

## Introduction

Tree transpiration plays an important role in the water balance of forest trees (Chang et al., 2014). Trunk sap flow is a direct indicator of tree transpiration that reflects the physiological characteristics of individual trees and is critical in maintaining hydraulic transport between the soil and atmosphere as well as in providing oxygen to thin-walled xylem cells and promoting nutrient uptake (Hubbart et al., 2007; Mcdowell et al., 2010). Studies have shown that plant sap flow is mainly influenced by meteorological factors and soil moisture (Yang et al., 2019; Zhao et al., 2021). Meteorological factors affect the water potential gradient between the boundary layer and the inner cavity of the leaves, thus affect the opening and closing of stomata (Chen et al., 2010). Kanalas et al. (2010) concluded that soil moisture is the fundamental factor affecting transpiration intensity and that if the soil moisture content is minimal, then the sap flow is minimal regardless of the meteorological factors driving it. The plant stem is the pathway through which soil water enters the leaves for transpiration (Tyree and Sperry, 1988), and changes in water tension in the trunk as the tree transpires cause microvariations in trunk diameter (Martínez-Vilalta et al., 2007). The shrinkage of the trunk indicates the depletion of water stored in the xylem, and the process of its diurnal variation indicates the sum of all external and internal conditions that affect the water relationships of the tree (Zweifel and Häsler, 2001). Therefore, the variations in trunk sap flow and stems at the daily scale may be able to jointly reflect the response of plant water use to changes in environmental conditions.

The thermal dissipation probe method (TDP) is a way of measuring stem sap flow velocity obtained by Granier after experiments, and it is widely used because of its relatively reliable measurement results, simple operation, and low cost (Liu et al., 2011; Molina et al., 2019). However, in recent years, many scholars have begun to question its accuracy, and it is generally believed that the measured values of trunk sap flow are lower than the actual values (Liu et al., 2012; Rana et al., 2019; Dix and Aubrey, 2021). Pasqualotto et al. (2019) found in *Corylus avellana* L. trunk sap flow that the original Granier’s formula underestimated the effective transpiration of the tree by 60%. Xie and Wan (2018) found that the errors in trunk sap flow measured by TDP ranged from 40 to 80% for *Quercus variabilis* Bl. and more than 80% for *Robinia pseudoacacia* L. It can be concluded that Granier’s original formula is not applicable to all tree species, and Smith and Allen (1996) recommended that the Granier formula coefficients be calibrated for each tree species. Currently, the *in vitro* stem weighing method is the main test method for sap flow correction. For example, the sap flow of *Pinus caribaea* Morelet, *Pinus sylvestris* L., *Fagus grandifolia* subsp., and *Tamarix ramosissima* Ledeb. were corrected by this method (Hultine et al., 2010; Steppe et al., 2010; Fan et al., 2018), and the fitting effect was better because the experimental conditions were controlled, reducing operational errors and external influences. However, the *in vitro* stem weighing method is performed under positive pressure, which is inconsistent with the vacuum state of trees under natural conditions and may also lead to the formation of embolism in the xylem and increased resistance to transport, resulting in an underestimation of the true values (Schenk et al., 2013; Fuchs et al., 2017). The whole-tree container weighing method is an accepted method but has problems such as invariable operation and higher labor intensity (Ma et al., 2021). However, the liquid level equilibrium (LLE) used in this paper is a new method of sap flow calibration formed by combining the whole-tree container method and the marsupial bottle water control method (Ladefoged, 1960; Roberts, 1977; Knight et al., 1981), and it is accurate in measurement and can be used as a base for simultaneous comparison and water consumption correction.

Currently, the main ecological problems in most areas of China are low per capita forest and green space, scarcity of water resources, and insecurity of ecological water (Gao et al., 2011). If we can accurately calculate the water consumption of forest trees and then realize precise irrigation, it is of certain importance for the effective use of ecological water resources in China. Therefore, in this paper, *Populus tomentosa* Carr. and *Salix babylonica* L., the main fast-growing timber forest species in northern China (Wang et al., 2018; Qu et al., 2022), were the objects of the research, and the changes in sap flow were continuously measured using TDP and the LLE methods and combined with the continuous changes in meteorological factors, soil moisture and radial direction of the trunk. The purpose of this paper is to answer the following questions: (1) changing characteristics and influencing factors of sap flow velocity, (2) verification of the original Grainer formulas, and (3) correction of the original Grainer formulas of *P. tomentosa* and *S. babylonica*, so as to reveal the transpiration water consumption pattern and water consumption capacity of the two species, and provide theoretical guidance for the accurate assessment of transpiration of major fast-growing timber forest species and the utilization and management of water resources, which is of great significance to improve the economic and ecological benefits of forest land in China.

## Materials and methods

### Study site

This experiment was conducted in the West Campus of Hebei Agricultural University in Baoding, Hebei Province, China (N38° 48′23″, E115° 24″58′, 20 m above sea level). The area is located in central Hebei Province and belongs to the warm temperate continental monsoon climate zone. The average annual temperature is 12°C, the average annual sunshine hours are 2,511 h. The average annual precipitation is approximately 575.9 mm, which is mainly concentrated in June—August, with the highest precipitation in July. The average annual wind speed is 1.8 m⋅s^–1^, and the average annual evaporation is approximately 1,430 mm.

The experiment period was from August to October of 2021. In April of 2021, nine *P. tomentosa* and *S. babylonica* specimens, each 3 years old, with healthy growth, without diseases or pests, and with straight trunks, were selected from the Science and Technology Park of Hebei Agricultural University and transplanted to the West Campus of Hebei Agricultural University and planted in large containers. The containers were 54.5 cm in diameter at the top, 41.0 cm in diameter at the bottom, and 41.0 cm in height, with a water supply and drainage port at the bottom. To make the drainage smooth, the barrel was padded with stones approximately 4 cm high. After transplanting, cultivated on soil containing nutrient substrate with reasonable water and fertilizer management, and after stabilizing growth, three sample trees with good and consistent growth of each *P. tomentosa* and *S. babylonica* were selected for the test (Table 1). At the beginning of the experiment, the containers were wrapped in all directions with radiation-proof films to prevent the evaporation of soil moisture and to reduce the damage of the containers by solar radiation. During the whole experiment, the containers were not always wrapped. Each experiment was conducted on 3–7 consecutive sunny days, and at the end of the experiment, the wrapped radiation-proof films were removed and the soil was allowed to breathe normally for about 3 days before the next experiment, thus avoiding the effect of long-term soil wrapping on the growth of the sample trees.

**TABLE 1:** Basic characteristics of sample trees.

Tree species	Number	Height (m)	DBH (cm)	Sapwood area (cm^2^)	Crown area (cm^2^)
*P. Tomentosa*	1	4.0	5.5	23.75	2.2
	2	3.9	5.2	21.23	1.9
	3	3.7	6.4	32.15	2.0
*S. Babylonica*	1	3.9	8.6	58.06	2.3
	2	3.7	8.9	62.18	3.4
	3	4.0	8.1	52.78	3.1

### Research methods

#### Measurement of sap flow velocity by thermal dissipation probe method

The sap flow velocity is measured by TDP. A set of thermal dissipation probes (AV-3665R, Rainroot Scientific Ltd., China) was installed in each tree, and the distance between the two probes is 10 cm. The basic principle is that two probes composed of thermal elements are inserted into the sapwood, and the probe inserted on top is heated using a constant current, while the probe below serves as a control. As the heat transfer from the sapwood increases with the stem flow velocity, more heat is removed from the heating probe, resulting in a temperature difference. The temperature difference between the two probes is greatest when the stem flow velocity is zero or very small (ΔTmax). 20 mm probes are used, powered by a 12 V rechargeable battery.

The installation was scheduled for July 2021. The probes were installed at 1.3 m above ground in the trees. First, the rough bark of the sample wood was scraped off at the probe mounting point, and then two vertical holes were drilled with a drill of a specific specification into which the probes were inserted. After the probes were inserted, they were clamped with a foam block and wrapped with insulating and antiradiation material after being fixed with tape. Finally, the probes were sealed with tape to prevent rainwater from entering. The whole stem flow velocity measurement system, including the stem flow meter, was composed of the TDP feedback line and data collector, which was used to collect and record the sap flow data automatically with a sampling frequency of 60 s and a data collection interval of 10 min. The sap flow velocity was calculated by Granier’s original formula (Granier, 1985) as follows:


$$F_{d}=0.0119\times K^{1.231}$$

$$F_{s}=F_{d}\times A_{s}$$

$$K=\left(\Delta T_{\max}-\Delta T\right)/\Delta T$$

Where *F*_*d*_ is the sap flow velocity (cm^3^⋅cm^–2^⋅S^–1^); *F_s_* is the sap flow (cm^3^⋅S^–1^); ΔTmax is the maximum temperature difference of the probes when the sap flow velocity is zero or very small. The weakest sap flow velocity of each day generally occurs in the early morning hours, and the Δ*T*_*max*_ is determined using the maximum temperature difference over a 3-day period in this study; Δ*T* is the temperature difference when sap flow velocity is occurring; *A*_*s*_ is the sapwood area of the sample (cm^2^).

The cumulative sap flow volume S (cm^3^) is:


$$S=\sum_{i=1}^{n}F_{\mathrm{si}}\times \Delta t$$

Where *n* is the number of collected samples; *F*_*si*_ is the sap flow (cm^3^⋅S^–1^) at the i-th collection; Δ*t* is the sampling interval time (s).

Results obtained with continuously heated TDP probes showed that the natural thermal gradient had a direct effect on the sap flow measurement with a large potential error, and the natural thermal gradient of more than 0.2°C was not negligible (Do and Rocheteau, 2002). Before conducting the experiment, we referred to the results of Ma (2020) for the sap flow measurement of the same tree species in the region and found that their natural thermal gradients were all below 0.2°C. Therefore, we neglected the effect of the natural thermal gradient in our experiment.

#### Measurement of transpiration by liquid level equilibrium method

**FIGURE 1:**
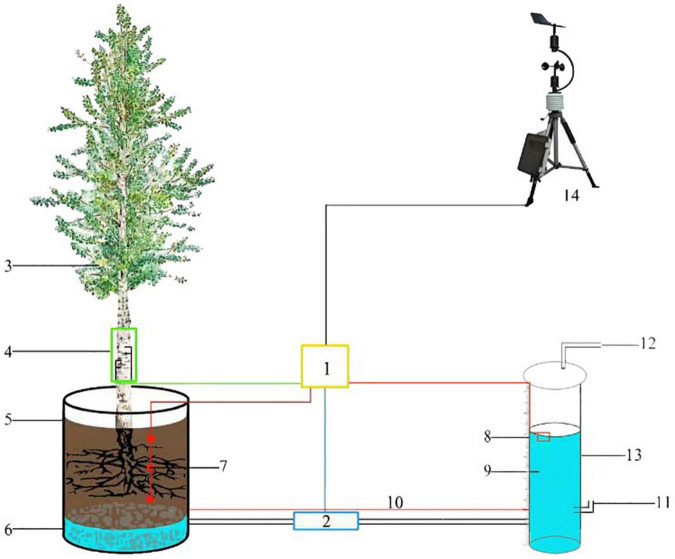
Schematic diagram of LLE method for measuring transpiration. 1, Data collector; 2, Switch; 3, Sample tree; 4, TDP; 5, Planting container; 6, Permeable matrix; 7, Soil moisture probe; 8, Scale line; 9, Water; 10, Equilibrium water level; 11, Intake pipe; 12, Exhaust pipe; 13, Marsupial bottle; 14, Weather station.

The transpiration is measured by LLE. Water levels of barrel-planted *P. tomentosa* and *S. babylonica* are recorded under continuous sunny, windless weather using a marsupial bottle, which is a bottle made of special glass (5 cm inner diameter, 5.6 cm outer diameter, 100 cm height). The upper end of the bottle has a vent, the lower end contains the vent at 8 cm above the ground and the water inlet at 3 cm from the ground, and the water inlet and the bottom of the barrel of the two drainage ports are connected with polyvinyl chloride (PVC) pipe to the end of the door valve to prevent the water from entering the barrel when filling the bottle. When filling the bottle, the door valve is closed, the upper exhaust port is opened, the air flows, and the atmospheric pressure is stable. When the water level reaches approximately 90 cm, the upper exhaust port is blocked, the door valve is opened, and the vacuum state is maintained in the bottle. When the water in the barrel reaches saturation, the height of the falling water level is recorded. The recording time was 6:00–20:00, and the water level change was recorded every half hour. To avoid the effect of sunlight on the bottle, it was wrapped with radiation-proof film. The difference in water level height between two consecutive records of the bottle multiplied by the bottom area is the transpiration of *P. tomentosa* and *S. babylonica* in half an hour (Figure 1). The marsupial bottle has the ability to control the water level and automatic replenishment, which allows the water level inside the planting container to be maintained at an appropriate level. Based on the water balance principle, the amount of water consumed in the marsupial bottle is equal to the amount of water consumed by the tree in the well-packed planting container.

#### Monitoring of soil moisture transport

A soil moisture monitor (TH302, Tang Hua Ltd., China) was used to monitor the change in soil moisture transport. Its working principle is to use probes to monitor the moisture in the soil in real time and then reflect the change in soil moisture content. The soil moisture monitoring probes were inserted into the two species, *P. tomentosa* and *S. babylonica*. The probes were inserted at soil depths of 10, 20, and 30 cm and were covered with soil. The other end of the probes were connected to a universal data collector, and the data collector was connected by Bluetooth to THHelper software on a cell phone. The data were recorded and stored at a time interval of 5 min.

#### Monitoring change of diameter at breast height

The change in diameter at breast height (DBH) was monitored using a trunk radial change recorder (DBL60, ICT International, Australia), which has a high resolution and is capable of measuring small changes in the stem to micrometer (μm) level accuracy. The principle of the operation is to monitor the stem change in real time using the relationship between the pressure and the sensor due to the radial variation in the trunk that exerts pressure on the fixed probe. The trunk radial change recorders were installed at the stem of each tree species (1.5 m above ground level) before the start of the formal experiment and were set to record and store data once every 30 min.

#### Monitoring of meteorological factors

During the test period, solar radiation (RA, W⋅m^–2^), relative air humidity (RH, %), atmospheric temperature (TA, °C), wind speed (WS, m⋅s^–1^), rainfall (Rain, mm) were automatically monitored by a weather station (RR-9170, Rainroot Scientific Ltd., China) located approximately 5 m from the sample site. The sampling interval was 10 min for recording and storage. The vapor pressure deficit (VPD, kPa) was calculated by the formula (Zhao et al., 2017):


$$\mathrm{VPD}=\left(1-\mathrm{RH}\right)\times 0.611e^{17.502\mathrm{TA}/\left(240.97+\mathrm{TA}\right)}$$

#### Determination of sapwood area

At the end of the test, the trunk core was drilled at a height of 1.3 m using a growth cone, and then the drilled core was dyed to observe the areas of sapwood and heartwood distribution, and the sapwood thickness was measured and recorded statistically to determine the sapwood area (Dang et al., 2020).

### Data statistics and analysis

The water consumption of the sample trees calculated by Granier’s original formula is the sap flow velocity, the water consumption recorded by LLE is the transpiration, and the transpiration divided by the sapwood area is the transpiration velocity. To facilitate verification and correction, the units are standardized to cm^3^⋅cm^–2^⋅s^–1^.

The transpiration measured by LLE was used as a benchmark to verify whether there was any error in the sap flow velocity measured by TDP. The temperature difference coefficient (K) measured by TDP was used as the horizontal axis, and the transpiration velocity measured by LLE was used as the vertical axis to establish calibration equations for *P. tomentosa* and *S. babylonica*. Then, the calibration equations of each tree species were used to test the sap flow of the corresponding tree species and to verify the validity of the equations. The data of the two species were combined to fit Granier’s correction formula for the bulkwood species, and the formula was tested against the sap flow velocity of the two species measured by TDP to verify the validity of Granier’s correction formula for bulkwood species.

Relative to the transpiration velocity measured by LLE, the coefficient of determination (*R*^2^) and the Willmott consistency index (D) were used to test the appropriateness of the calibration formulas, the root mean square error (RMSE) and the mean absolute error (MAE) were used to test the accuracy of the calibration formulas and transpiration velocity, and the mean bias error (MBE) was used to determine the deviation of the calibration formulas and transpiration velocity.


$$D=1-\frac{\sum_{i=1}^{n}{\left(E_{i}-O_{i}\right)}^{2}}{\sum_{i=1}^{n}{\left(\left|E_{i}-\bar{O}\right|+\left|O_{i}-\bar{O}\right|\right)}^{2}}$$

$$\mathrm{RMSE}={\left[n^{-1}\sum_{i=1}^{n}{\left(E_{i}-O_{i}\right)}^{2}\right]}^{0.5}$$

$$\mathrm{MAE}=\frac{1}{n}\sum_{i=1}^{n}\left(E_{i}-O_{i}\right)$$

$$\mathrm{MBE}=\frac{\sum_{i=1}^{n}\left(E_{i}-O_{i}\right)}{n}$$

Where, *n* is the number of observations; E_*i*_ is the sap flow velocity of the calibration equations; *O*_*i*_ is the transpiration velocity, and $\bar{O}$ is the average of the transpiration velocity. The closer *R*^2^ is to 1, the better the fitted model; the *D* index is close to 1, and the closer *RMSE*, *MAE*, and *MBE* are to 0, indicating that the transpiration velocity is in good agreement with the sap flow velocity of the calibration equations (Abbas et al., 2011).

Microsoft Excel 2016 was used to organize and calculate the original data, and SPSS 24.0 was applied to analyze the correlation between environmental factors and sap flow velocity.

## Results and analysis

### Characteristics of changes in sap flow velocity and influencing factors

#### Characteristics of changes in sap flow velocity

**FIGURE 2:**
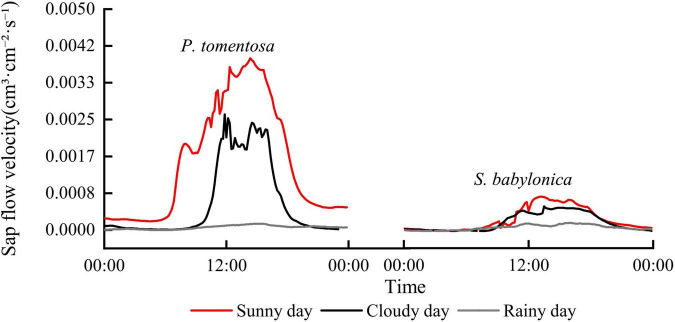
The changes of sap flow velocity of *P. tomentosa* and *S. babylonica* under different weather conditions.

Figure 2 shows that the daily changes in sap flow velocity of *P. tomentosa* and *S. babylonica* under typical sunny, cloudy and rainy conditions showed an increasing and then decreasing pattern, and the sap flow velocity was sunny > cloudy > rainy. The sap flow velocity of *P. tomentosa* showed a “single-peak” pattern of change on sunny days, a “double-peak” pattern on cloudy days, and fluctuated very little on rainy days. The sap flow velocity of *S. babylonica* showed a “single-peak” pattern on sunny and cloudy days, and it was also very small on rainy days.

#### Response of sap flow velocity to meteorological factors

The correlations between the sap flow velocity of *P. tomentosa* and *S. babylonica* and meteorological factors were analyzed on sunny, cloudy and rainy days (Table 2), and the results showed that the sap flow velocity of *P. tomentosa* was highly significantly and positively correlated with TA, RA, and VPD, highly and negatively correlated with RH on sunny, cloudy and rainy days, the overall correlation between sap flow velocity and meteorological factors was weak on rainy days. The sap flow velocity of *S. babylonica* was highly significantly and positively correlated with TA, RA, WS, and VPD, highly significantly and negatively correlated with RH on sunny and cloudy days, and it was also weakly correlated with each meteorological factor on rainy days.

**TABLE 2:** Correlations between sap flow velocity of *P. tomentosa* and *S. babylonica* and meteorological factors.

Species	Weather	TA	RH	RA	WS	VPD
*P. tomentosa*	Sunny day	0.875**	-0.902**	0.823**	0.860**	0.958**
	Cloud day	0.917**	-0.887**	0.814**	0.945**	0.900**
	Rainy day	0.604**	-0.434**	0.572**	-0.151	0.529**
*S. babylonica*	Sunny day	0.927**	-0.899**	0.696**	0.889**	0.953**
	Cloud day	0.895**	-0.904**	0.741**	0.835**	0.917**
	Rainy day	0.197*	0.518**	0.547**	0.636**	-0.480**

*P < 0.05, **P < 0.01.

To further clarify the main meteorological factors affecting *P. tomentosa* and *S. babylonica* under different weather conditions, stepwise regression analysis was used to establish regression models with TA, RH, RA, WS, and VPD, and the factors entering the models were the main influencing factors. The regression equations of sap flow velocity and meteorological factors on sunny, cloudy and rainy days were as follows:

*P. tomentosa* (sunny days) = 1.137VPD+0.001RA+ 0.015RH - 1.916 *R*^2^ = 0.926

*P. tomentosa* (cloudy days) = 0.014WS+0.037TA+0.001RA - 0.009RH+0.13 *R*^2^ = 0.967

*P. tomentosa* (rainy days) = 0.031TA+0.743VPD-0.001WS - 0.379 *R*^2^ = 0.580

*S. babylonica* (sunny days) = 0.497VPD+0.013RH- 0.025TA - 0.746 *R*^2^ = 0.954

*S. babylonica* (cloudy days) = 0.140VPD - 0.092 *R*^2^ = 0.899

*S. babylonica* (rainy days) = 0.001WS+0.062RH+0.040TA - 0.001RA+2.049VPD - 6.864 *R*^2^ = 0.863

From the above Equations, it can be seen that the main influencing factors of sap flow velocity of *P. tomentosa* on sunny days were VPD, RA, and RH; on cloudy days they were WS, TA, RA, and RH; on rainy days they were TA, VPD, and WS; the main influencing factors of sap flow velocity of *S. babylonica* on sunny days were VPD, RH, and TA; on cloudy days they were VPD; and on rainy days they were WS, RH, TA, RA, and VPD.

#### Relationship between sap flow velocity and diameter at breast height

**FIGURE 3:**
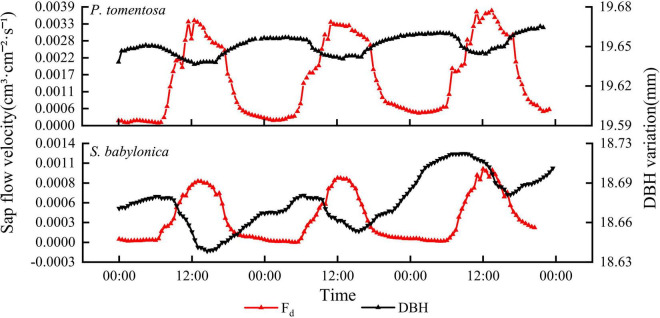
The changes of sap flow velocity and DBH of *P. tomentosa* and *S. babylonica.*

As seen in Figure 3, the sap flow velocity and DBH changes of both *P. tomentosa* and *S. babylonica* showed opposite trends at the daily scale. During the daytime, with increasing temperature and solar radiation, transpiration of the trees was enhanced, and the sap flow velocity was accelerated. The water stored in the trunk during the previous night might be utilized preferentially, so the water in the trunk was reduced continuously, leading to the shrinking of the stem, and the sap flow velocity increased continuously at this stage. Later, as the temperature and solar radiation decreased, transpiration also decreased gradually, the sap flow velocity began to show a decreasing trend, the stem began to expand slowly until the temperature and solar radiation increased, the sap flow velocity increased, and the stem contracted again.

As seen in Table 3, the DBH microvariation and TA, RH, RA, and VPD of *P. tomentosa* and *S. babylonica* were all highly significantly correlated. Both were highly significantly negatively correlated with TA, WS, and VPD and highly significantly positively correlated with RH.

**TABLE 3:** Correlation coefficients between DBH microvariation and meteorological factors of *P. tomentosa* and *S. babylonica.*

Species	TA	RH	RA	WS	VPD
*P. tomentosa*	-0.593**	0.360**	-0.664**	-0.745**	-0.548**
*S. babylonica*	-0.608**	0.736**	-0.288**	-0.429**	-0.737**

**P < 0.01.

#### Response of sap flow velocity to soil water transport

**FIGURE 4:**
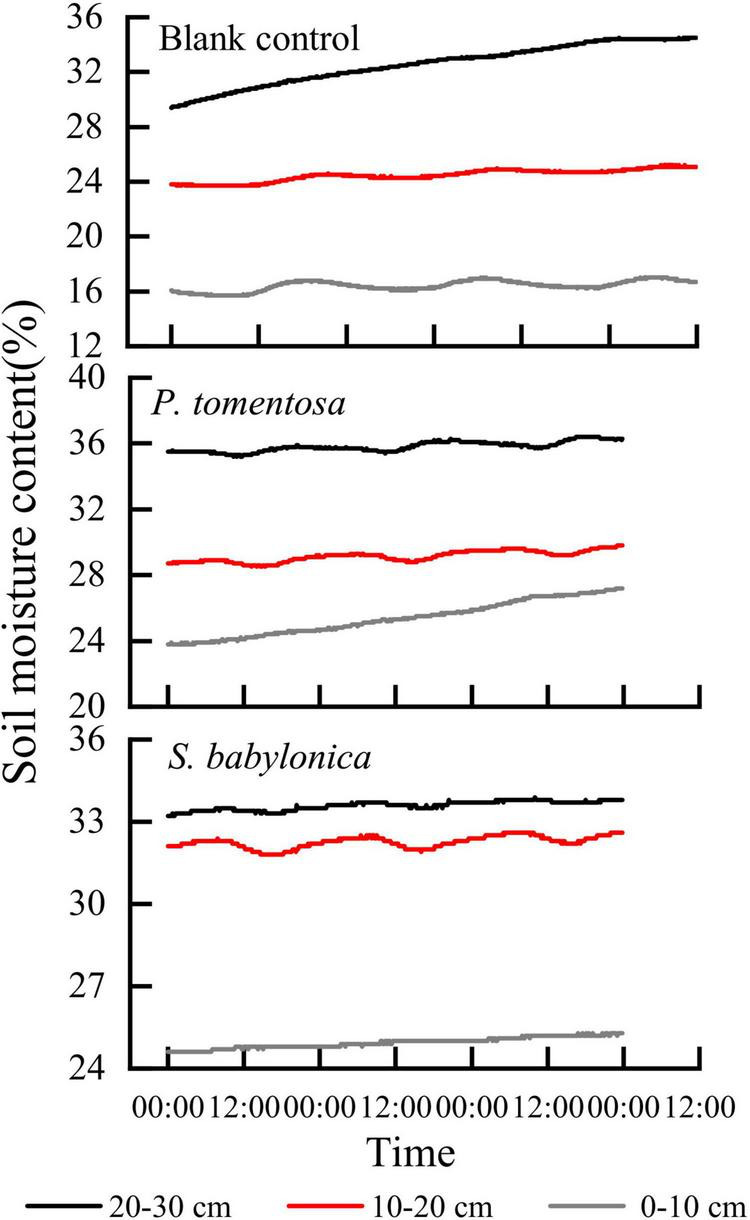
The changes of soil water content in blank control, *P. tomentosa* and *S. babylonica* at different soil depths.

A container full of soil without trees planted was used as a blank control, and the marsupial bottles continuously supplied water to the *P. tomentosa* and *S. babylonica* planted in the containers and the blank control. It can be seen from Figure 4 that the higher the depth of the blank experimental soil layer is, the greater the soil water content, and the soil water content at different depths showed an increasing trend, and the greater the depth of the soil layer is, the more obvious the trend of soil water content change. As soil capillary water was transported upward under the action of matrix potential and gravitational potential, its rising rate gradually decreased with time under continuous water supply. For *P. tomentosa* and *S. babylonica*, the lower the depth of the soil layer is, the lower the soil water content. Water was transported from bottom to top, and its soil water content was lower as it was closer to the surface soil. The trend of soil water content changes at three different depths was not the same, which might be caused by the large absorption of surrounding soil water by the roots. The soil water content was highest at depths of 20–30 cm, and the trend of change was not obvious, indicating that there might be a small amount of roots distributed in this layer, while the trend of soil water content at depths of 10–20 cm was obvious and similar to the trend of sap flow velocity, which indicated that the roots of *P. tomentosa* and *S. babylonica* may be mainly distributed in this soil layer. The upward trend of the change of soil water content at 0–10 cm depth may be due to sufficient water to make the soil water continuously transported upward, even though the phenomenon of water absorption by the roots existed at this soil layer, but in general it did not affect the continuous rise, and in the process of rising and constantly approaching the soil water content at 10–20 cm, it could finally reach a relative equilibrium state.

**FIGURE 5:**
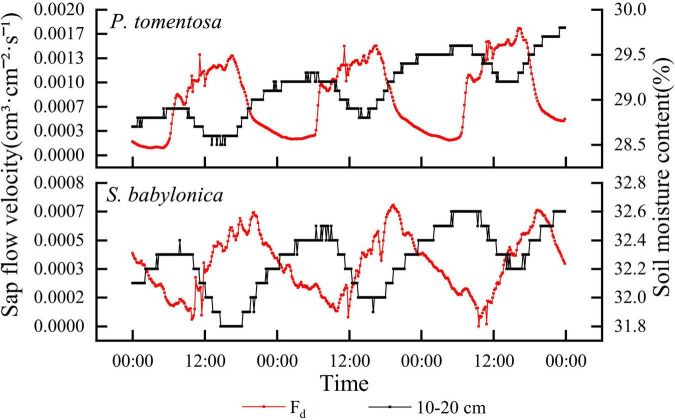
The changes of sap flow velocity and soil water content of *P. tomentosa* and *S. babylonica*.

The correlations between the sap flow velocity and soil water content at different depths were determined for *P. tomentosa* and *S. babylonica*, and the results showed that the sap flow velocity of both species was highly significantly negatively correlated with the soil water content at 10–20 cm (*P* < 0.01). Figure 5 shows that the sap flow velocity of *P. tomentosa* and *S. babylonica* showed an opposite trend with the soil water content at 10–20 cm. As the roots of trees may be widely distributed at this depth, with the increase in transpiration, the trees gradually absorb a large amount of water, and when the root absorption rate is greater than the water transport rate, the soil water content begins to decline; when the transpiration rate gradually decreases, the root absorption is smaller than the soil water transport rate, and then the soil water content gradually increases.

### Validation and correction of the original Granier’s formula

#### Validation of the original Granier’s formula

**FIGURE 6:**
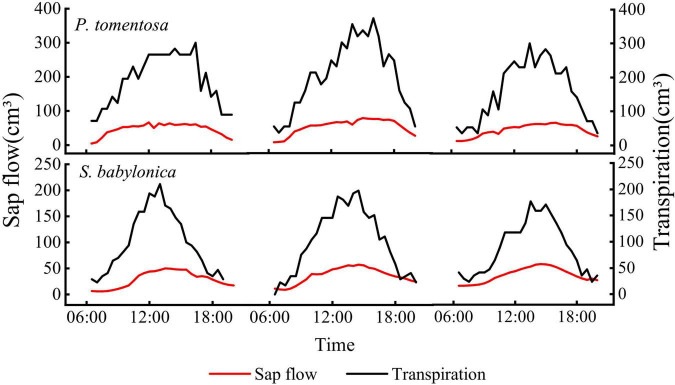
Daily changes of transpiration and sap flow of *P. tomentosa* and *S. babylonica*.

TDP was used to measure the sap flow, and LLE was used to simultaneously measure transpiration. The sap flow measured by TDP of the two species was positively correlated with the transpiration measured by LLE (*P* < 0.01), showing a consistent pattern of variation, but the transpiration was significantly greater than the sap flow (Figure 6), indicating that the TDP-measured sap flow underestimated the true values.

**FIGURE 7:**
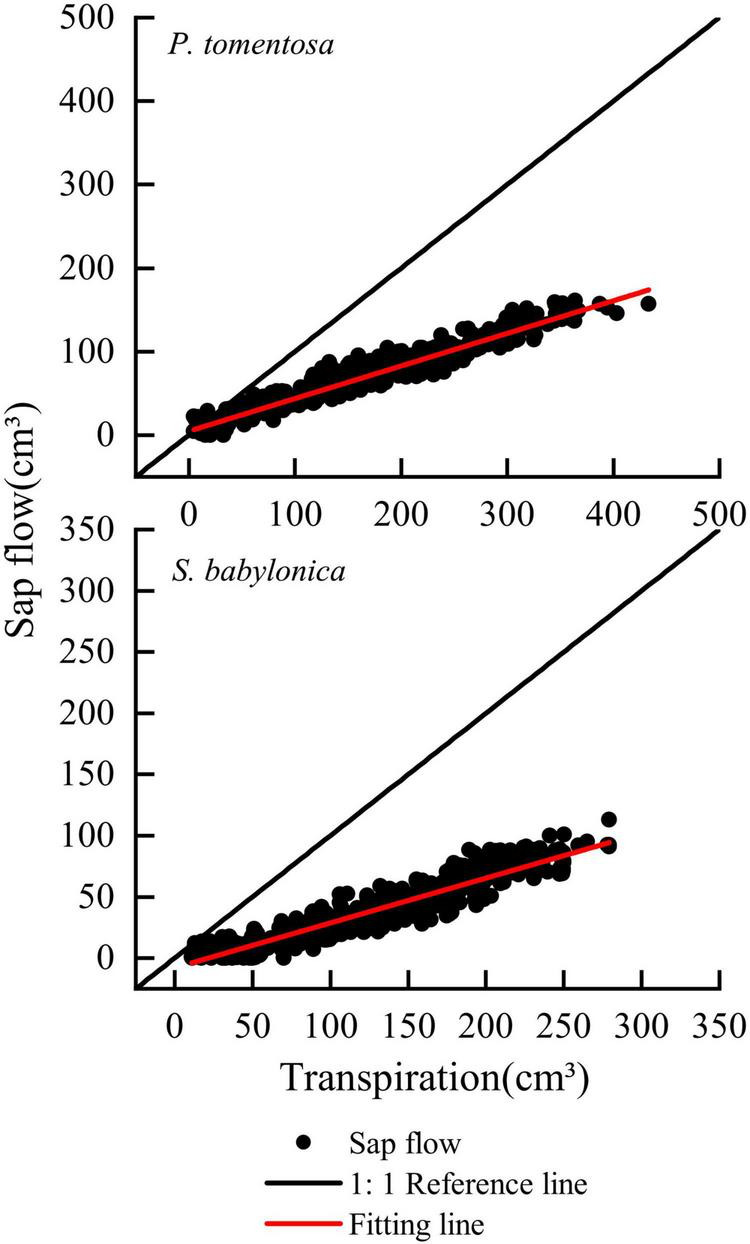
Validation of the original Granier’s formulas of *P. tomentosa* and *S. babylonica.*

The transpiration and sap flow of the two species were linearly fitted separately (Figure 7). The sap flow of *P. tomentosa* and *S. babylonica* were both smaller than the transpiration, with error values of -60.96% and -63.37%, respectively. Therefore, it is necessary to correct the original Granier’s formulas.

#### Correction of the original Granier’s formula

**FIGURE 8:**
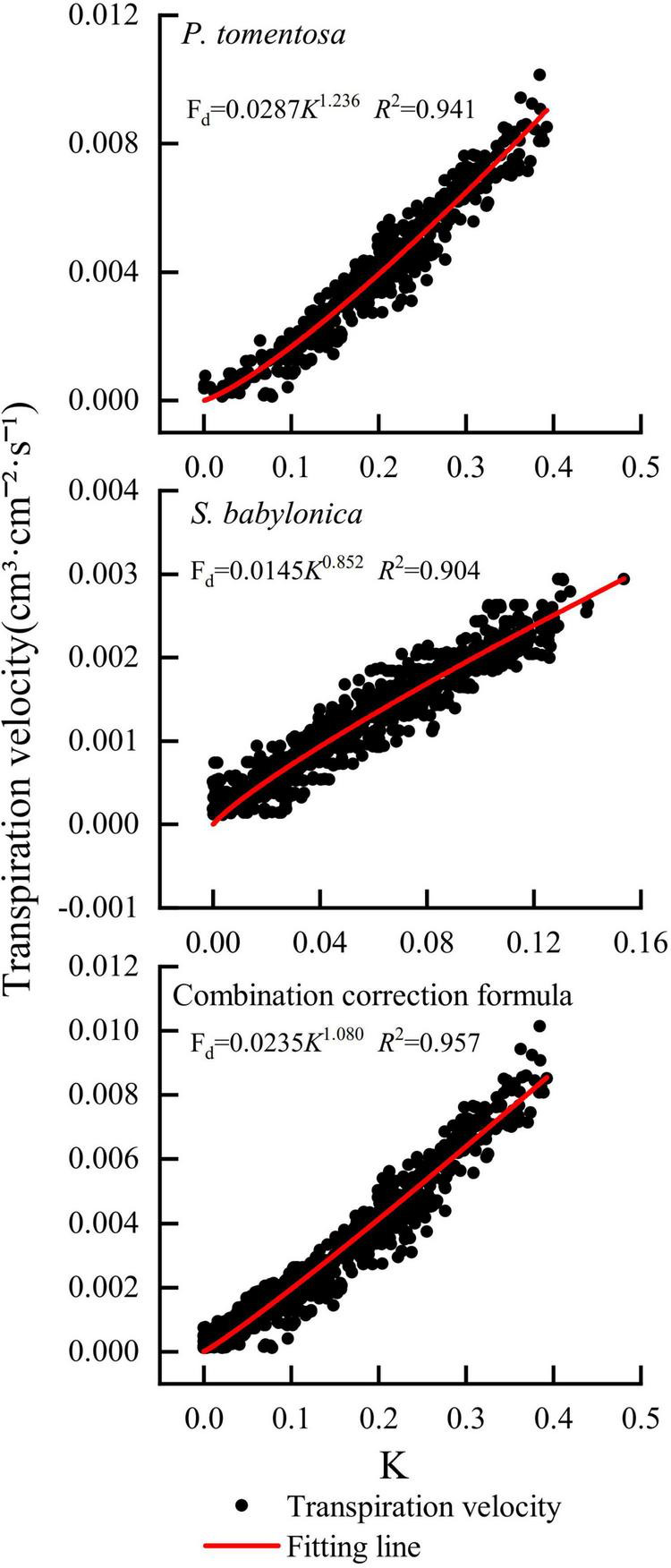
Correction formulas for *P. tomentosa* and *S. babylonica*.

The correction equations were obtained by fitting a power function with the TDP-measured K as the horizontal coordinate and the LLE-measured transpiration rate as the vertical coordinate. The specific correction equations for *P. tomentosa* and *S. babylonica* were F_*d*_ = 0.0287*K*^1.236^ (*R*^2^ = 0.941) and F_*d*_ = 0.0145*K*^0.852^ (*R*^2^ = 0.904), respectively. Compared with Granier’s original formula F_*d*_ = 0.0119*K*^1.231^, the coefficient α of the correction formula for *P. tomentosa* (0.0287) is larger than that of Granier (0.0119), and the correction coefficient β (1.236) is similar to that of Granier (1.231); the coefficient α (0.0145) of the correction formula for *S. babylonica* is larger than that of Granier (0.0119), and the correction coefficient β (0.852) is smaller than that of Granier (1.231). The K values and transpiration rates of *P. tomentosa* and *S. babylonica* were integrated to establish the combined correction equation: F_*d*_ = 0.0235*K*^1.080^ (*R*^2^ = 0.957). Compared with Granier’s original formula F_*d*_ = 0.0119*K*^1.231^, the coefficients α (0.0235) and β (1.080) of the combined correction formula were both different from the coefficients of Granier’s original formula (Figure 8).

#### Validation of Granier’s correction formulas

**FIGURE 9:**
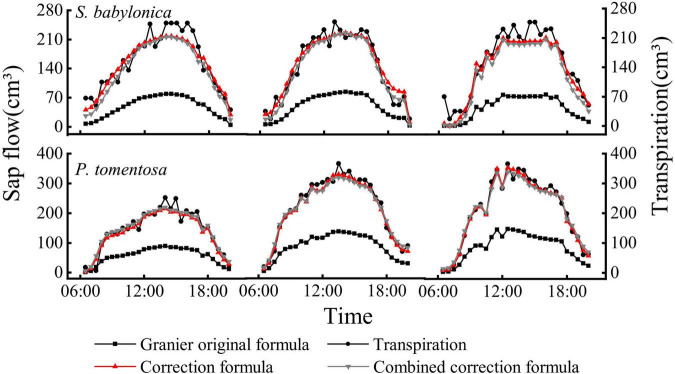
Daily variations of transpiration and sap flow of *P. tomentosa* and *S. babylonica*.

There were significant positive correlations between the sap flow measured by Granier’s original formula, specific correction formulas, combined correction formula, and transpiration measured by LLE of *P. tomentosa* and *S. babylonica* (*p* < 0.01); however, the sap flow calculated by Granier’s original formula was obviously lower than that calculated by the other three methods (Figure 9). Compared with Granier’s original formula, both the specific correction formulas and the combination correction formula were closer to the transpiration value measured by LLE.

**FIGURE 10:**
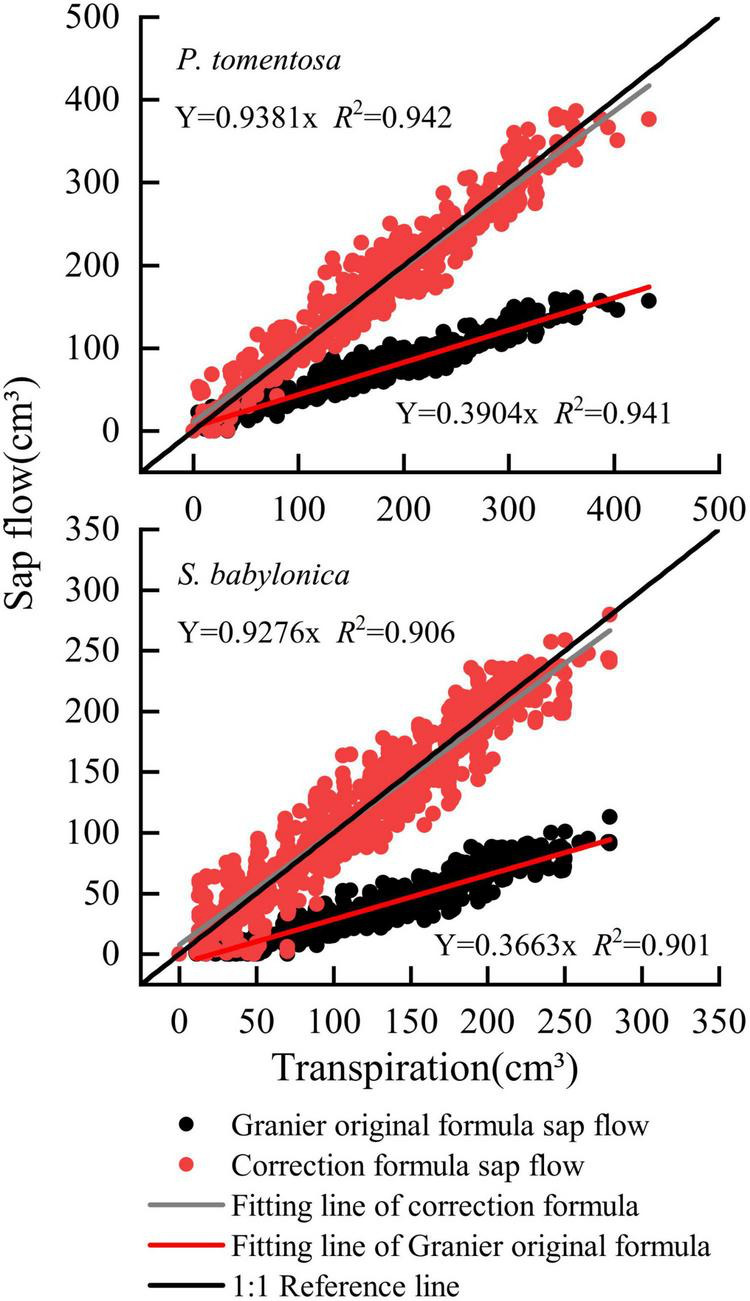
Verifying the correction formulas of *P. tomentosa* and *S. babylonica*.

The sap flow calculated by Granier’s original formula and specific correction formula and the transpiration measured by LLE of *P. tomentosa* and *S. babylonica* were fitted linearly (Figure 10). The results showed that the sap flow calculated by the specific correction formulas was closer to the reference line of 1:1, while that calculated by Granier’s original formula was obviously lower. Compared with those of transpiration, the errors of sap flow calculated by the specific correction formulas were 6.18% and 5.86%, respectively, and the errors were obviously reduced.

**FIGURE 11:**
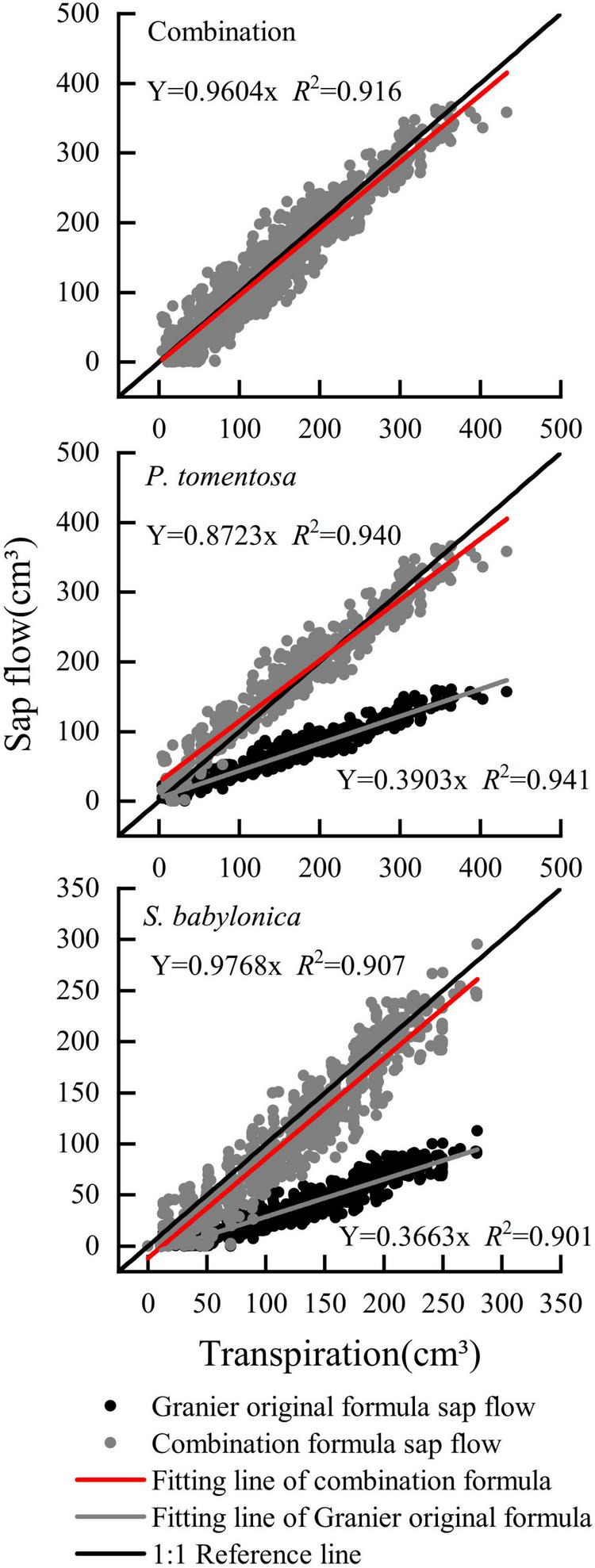
Verifying the combination formula of *P. tomentosa* and *S. babylonica.*

As shown in Figure 11, the overall sap flow calculated by the combined correction formula for *P. tomentosa* and *S. babylonica* was closer to the 1:1 reference line, with an error of -3.97%. The errors of the combined correction formula for *P. tomentosa* and *S. babylonica* were -12.76% and -2.32%, respectively, which was more accurate.

To further verify the accuracy of the specific and combined correction formulas for *P. tomentosa* and *S. babylonica*, the validity of the formulas was tested for deviations from the true transpiration rates using the D-index, RMSE, MAE, and MBE (Table 4). The combined correction formula and specific correction formulas of *P. tomentosa* and *S. babylonica* were both valid and similar, with the transpiration rates measured by LLE.

**TABLE 4:** Effectiveness of *P. tomentosa* and *S. babylonica* in calculating sap flow velocity by Granier’s original formula, specific correction formulas and combined correction formula.

Species	Correction formula	D index	RMSE (cm^3^⋅s^–1^)	MAE (cm^3^⋅s^–1^)	MBE (cm^3^⋅s^–1^)
*P. tomentosa*	Granier’s original formula	0.620	0.002699	-0.002345	-0.002345
	Specific correction formula	0.985	0.000524	0.000001	0.000001
	Combined correction formula	0.981	0.000567	0.000126	0.000126
*S. babylonica*	Granier’s original formula	0.580	0.001001	-0.000897	-0.000897
	Specific correction formula	0.975	0.000213	-0.000011	-0.000011
	Combined correction formula	0.964	0.000266	-0.000153	-0.000153

## Discussion

### Characteristics of changes in sap flow velocity and influencing factors

The daily patterns of sap flow velocity vary significantly under different weather conditions. Most studies concluded that the sap flow velocity showed a single-peaked or a double-peaked curve on sunny days (Zhu et al., 2013). Yu (2010) showed that the sap flow velocity of *Robinia pseudoacacia* L. showed a single-peaked curve on sunny days and a double-peaked curve on cloudy days, which is consistent with the results of this study on *P. tomentosa.* In contrast, the sap flow velocity of *S. babylonica* showed a single-peaked curve on both rainy and sunny days, which may be greatly related to the selection of tree species. Most studies concluded that sap flow velocity had no obvious pattern of change on rainy days and that it was greater on sunny days than on rainy days (Li et al., 2019; Wang, 2020). In this study, the sap flow velocity of *P. tomentosa* and *S. babylonica* changed minimally and it was at the lowest velocity during rainy days, which was mainly due to the high relative humidity of the air during rainy days. The vapor pressure deficit between the inside and outside of the leaves was smaller on rainy days, and the rainfall could lead to the closure of leaf stomata, thus showing a greater inhibitory effect on tree transpiration (Cui et al., 2020).

Various environmental factors, such as the biological structure of trees and soil moisture content, exert constraints on the sap flow velocity (Yang et al., 2013). Studies have shown that sap flow velocity was significantly and positively correlated with RA, TA, and WS and significantly and negatively correlated with RH (Granier et al., 1996). As with the results of this study, the sap flow velocity of both *P. tomentosa* and *S. babylonica* under sunny and cloudy days was highly significantly and positively correlated with TA, RA, WS, and VPD and highly and negatively correlated with RH, and the overall correlation between sap flow velocity and meteorological factors was weak during rainy days, indicating that sap flow changes increased with TA, RA, and WS and decreased with RH. However, the main environmental factors affecting the trunk sap flow velocity of the two species in the same environment were different in this study, which may be related to the physiological and biochemical characteristics of the trees themselves (Li et al., 2019). Gong et al. (2011) pointed out that although environmental factors had an extremely important influence on tree trunk sap flow, species variability was also one of the important factors that cannot be ignored in sap flow variation, confirming the complexity and relativity of the mechanisms affecting and regulating transpiration water consumption in trees (Li et al., 2017). Studies have shown that more than 90% of the water absorbed by the soil is dissipated to the atmosphere in the form of transpiration (Wang et al., 2012). Ma et al. (2017) concluded that the normal growth of *Populus beijingensis* W. Y. Hsu was affected when it was subjected to water stress, and Wang et al. (2020) found that the transpiration rate of *Betula platyphylla* Suk. was accelerated when the soil water content was relatively sufficient; when the soil water was deficient, the stomata were closed or incompletely opened to adapt to the water stress, and the transpiration rate was reduced. Unlike the results of this study, the soil water content at 10–20 cm in *P. tomentosa* and *S. babylonica* was more variable, and the change in soil water content at this layer and sap flow velocity were highly significantly negatively correlated, this may be because this experiment was conducted in containers, and the marsupial bottles were constantly supplying sufficient water. The soil moisture meter monitored the change in soil moisture at the microscopic level, and at the macroscopic level, it was always at saturation. Transpiration leads to the depletion of stored water in sapwood tissues, some contraction and expansion of stems occurs at the daily scale, and water reserves in the stem contribute to transpiration and play an important role in optimizing water transport by buffering the extremes of water depletion (Zweifel et al., 2001). Through studies on *Populus hopeiensis* Hu et Chow, *Pinus tabulaeformis* Carr., *Quercus liaotungensis* Blume, and *Robinia pseudoacacia* L. (Liu et al., 2018, 2021), it was found that sap flow velocity showed an inverse pattern of variation with daily changes in stems, which is the same as the results of this experiment. Stem microvariation was considered to be correlated with meteorological factors such as TA, VPD, TA, and rain (Drew and Downes, 2009; Ehrenberger et al., 2012), which is similar to the conclusions reached in the present experiment, further indicating that stem daily variation is a marker indicating the water status of plants. It was concluded that the coordinated response of transpiration, stem water storage dynamics and tree growth-based water use efficiency allows trees to cope with seasonal and interannual drought conditions (Sánchez-Costa et al., 2015). Correlation analysis revealed that *P. tomentosa* sap flow velocity was highly significantly negatively correlated with stem change (*P* < 0.01) and significantly negatively correlated with soil water content (*P* < 0.05), and soil water content was highly significantly positively correlated with stem change (*P* < 0.01). *S. babylonica* sap flow velocity was highly significantly negatively correlated with both stem change and soil water content (*P* < 0.01), and stem change was significantly positively correlated with soil water content (*P* < 0.05), which showed that there was a close relationship between sap flow velocity, stem microvariation and soil water content.

### Validation and correction of the original Granier’s formula

Granier’s original formula, which is one of the core elements of the heat dissipation sap flow determination technique, was constructed based on the calibration results of three species of *Pseudotsuga menziesii* (Mirb.) Franco, *Pinus nigra* Arn. and *Quercus pedunculata* Ehrh. and extended to other tree species for application (Granier, 1985). The formula was considered to be applicable to all tree species. However, in recent years, some scholars have studied the results of *Quercus alba* L., *Corylus avellane* Linn., *Fagus grandifolia* subsp., *Robinia pseudoacacia* L., *Quercus variabilis* Bl., *Tamarix ramosissima* Ledeb. and *Chimononthus praecox* (L.) Link and noted that there were cases of underestimation or overestimation of the true values of sap flow with relative errors ranging from -80 to 9% (Hultine et al., 2010; Steppe et al., 2010; Sun et al., 2012; Xie and Wan, 2018). In this study, the errors of *P. tomentosa* and *S. babylonica* were -60.96% and -63.37%, respectively. TDP measurement errors are mainly due to the biological and physical characteristics of trees, such as the sapwood area of Granier’s original formula, which requires the measurement of cross-sectional area without damaging the tree, possibly resulting in errors in determining the sapwood area (Jia et al., 2020; Ma et al., 2020). The length of sapwood conduits, the size of conduit diameter, and the number of grain pores, which affect the water transfer efficiency of conduits (Baas, 1976; Tyree and Zimmermann, 2013; Li, 2018). The differences in hydraulic conductivity of wood in different orientations, and the non-uniform distribution of trunk sap flow in radial depth caused by differences in xylem structure, which can also cause measurement errors (Becker, 1996; Cermak and Nadezhdina, 1998; Nadezhdina et al., 2002; Tateishi et al., 2008). The determination of the maximum temperature difference ΔTmax in Granier’s original formula, which may also have some errors that may vary with temperature and sap flow velocity (Lu et al., 2004). The fact that as the measurement time increases, the thermal damage to the trunk caused by the TDP heating probes increases, which may also have an impact on the accuracy of the measurement (Wiedemann et al., 2016). Thus, the effects of wood anatomy and the surrounding environmental factors on sap flow should be further investigated to reveal the causes of errors.

Because of the varying errors in the determination of trunk sap flow by TDP, many researchers have calibrated the original Granier’s formula coefficients. Sun et al. (2012) used the calibration coefficients for the calculation of sap flow in *Populus deltoides* Marsh. (α:0.0121, β:1.141), *Quercus alba* L. (α:0.0128, β:1.470), *Ulmus americana* L. (α:0.0272, β:2.572), *Pinus echinata* Mill. (α:0.0101, β:1.303), and *Pinus taeda* L. (α:0.0097, β:1.336) to reduce the errors of these five tree species to approximately 4% and the error for *Liquidambar styraciflua* L. (α:0.0124, β:1.115) to 8%. Rubilar et al. (2017) quantified the sap flow of 2-year-old *Eucalyptus globulus* L. using Granier’s original formula and further improved the sap flow determination using the calibrated parameters α = 0.0385 and β = 1.245. In this study, by verifying and correcting the original Granier’s formula for *P. tomentosa* and *S. babylonica*, the specific correction equations were obtained as F_*d*_ = 0.0287*K*^1.236^ (*R*^2^ = 0.941) and F_*d*_ = 0.0145*K*^0.852^ (*R*^2^ = 0.904), respectively, and the combined correction equation formula was F_*d*_ = 0.0235*K*^1.080^ (*R*^2^ = 0.957). For both the specific correction equations and the combined correction equation, the coefficients were all different from Granier’s original equation, and both fit better. It can also be reflected that both species belong to bulk-porous timber species, and the wood anatomical structures are relatively homogeneous in both, which makes them suitable for the development of a generalized calibration formula for bulk-porous timber. In contrast, the ring-porous and nonporous species need further calibration measurements because there are large differences in moisture transport structures for different species, as Bush et al. (2010) pointed out that the TDP method had a greater error rate for true sap flow with ring-porous wood, while Sun et al. (2012) also found that TDP underestimated sap flow more for bulk-porous wood (34–55%) than for ring-porous wood (9–15%). It is evident that the wood anatomy of different species can significantly affect the accuracy of TDP (Lu et al., 2004). In future studies, to improve the estimation accuracy, it can be combined with methods such as the lysimeter method (Tfwala et al., 2018) and high-precision electronic potometer method (Pasqualotto et al., 2019) to construct combined calibration equations (Bleby et al., 2004; Fuchs et al., 2017) by stand type or climatic zone, classified according to bulk-porous, ring-porous and nonporous material, which is more generalizable.

## Conclusion

The sap flow velocity of *P. tomentosa* and *S. babylonica* under different weather conditions showed different curves. Meteorological factors and soil moisture content are the driving factors of sap flow velocity of both species. Sap flow velocity, soil water content and stem daily variation together reflected the water use dynamics of *P. tomentosa* and *S. babylonica.*

Compared to that measured by the LLE method, the sap flow measured by Granier’s original formula for *P. tomentosa* and *S. babylonica* was severely underestimated. We established and validated the specific correction formulas and the combined correction formula for both species, and the errors of sap flow calculated by the specific correction formulas for both species were -6.18% and -5.86%, and those calculated by the combined correction formula were -12.76% and -2.32%, respectively. Thus, the accuracy of the established specific correction formulas and the combined correction formula for estimating the sap flow of *P. tomentosa* and *S. babylonica* were both considerable.

## Data availability statement

The raw data supporting the conclusions of this article will be made available by the authors, without undue reservation. The raw data for this study can be found in Supplementary material 1.

## Author contributions

YL drafted, revised the manuscript, and contributed to data analysis. HZ participated in the experiments and contributed to data analysis. CD revised the manuscript and performed the maintenance of the instruments. CM and BL designed, coordinated the study and revised the manuscript. All authors contributed to the article and approved the submission.

## Abbreviations

LLE: liquid level equilibrium method
TDP: thermal diffusion probe method
F_*d*_: flow velocity
A_s_: area.
